# Qualitative and semi‐quantitative primary and secondary transfer of metal traces by human touch detected using SEM–EDS technique: A pilot study

**DOI:** 10.1111/1556-4029.70285

**Published:** 2026-02-11

**Authors:** Barbara Bertoglio, Sofia Bodini, Alberto Amadasi, Giacomo Govoni, Giovanni Cecchetto, Silvia D. Visonà

**Affiliations:** ^1^ Department of Public Health, Experimental and Forensic Medicine, Unit of Legal Medicine and Forensic Sciences University of Pavia Pavia Italy; ^2^ Institute of Forensic Medicine of Brescia University of Brescia Brescia Italy; ^3^ Institute of Legal Medicine and Forensic Sciences Charité‐Universitäts Medizin Berlin Berlin Germany; ^4^ Fondazione IRCCS Policlinico San Matteo Pavia Italy

**Keywords:** crime reconstruction, energy‐dispersive spectroscopy, metal particles, primary transfer, scanning electron microscopy, secondary transfer

## Abstract

The present pilot study aimed to assess the transfer of metal residues on fingertips after contact with different items (primary transfer) as well as the transfer of metal residues by human fingertips from metal objects to different surfaces (secondary transfer). In the first part of the experiment, one volunteer touched 10 metal items with different characteristics and, after each touching, a single fingermark was collected using a stub. In the second part, a volunteer touched each of the objects and then another surface (glass, plastic, or the skin of a second volunteer). After each touching, a sample was collected using a stub from the touched surface. Then, the stubs were observed using a scanning electron microscope, and metal traces were quantified and analyzed using energy‐dispersive spectroscopy. The analysis revealed a strong presence of traces in the first touch samples: in all of them, more than 50% of the examined fields presented traces composed of the same elements revealed on the objects. The highest secondary transfer was observed on plastic, followed by skin and glass. Overall, the quantity of transferred particles is higher for metal items with a rough surface compared with smooth ones. In conclusion, this work provides experimental data that can be useful in a forensic setting, in order to link objects, actions, and people, and that can help in the difficult task of reconstructing the dynamics of a crime.

**Highlights**

- Systematic scanning electron microscope equipped with energy‐dispersive spectroscopy (SEM–EDS) analysis reveals both primary and secondary touch transfer of metal particles.
- Plastic retains more metal particles than skin or glass, depending on the recipient surface.
- Rough metal items yield greater secondary transfer of particles than smooth‐surfaced items.
- Particle distribution and aggregation vary with object features and the specific type of contact.

## 1 INTRODUCTION

According to Locard's exchange principle, “*Every contact leaves a trace”* [1]. For “primary transfer” we mean the transfer of particles on the hands of the person who touched a given item, while the term “secondary transfer” refers to the transfer of traces on a second surface which is touched after handling a given item. Most existing studies about particle transfer focus on fibers and hair [2]. Very few studies investigated the transfer of metal particles by human touching. French et al. (2012) [2] used an ultraviolet powder as a proxy for trace evidence in three different experimental scenarios, confirming the occurrence of secondary, tertiary, and quaternary transfer from a “primary source” through intermediaries. As the secondary transfer goes on, the primary source is depleted of traces, but the material is not lost but redistributed within the forensic setting (some objects were found to serve as “reservoirs” of trace materials). The same study demonstrated that the transfer can take place up to 5 h after the initial touching. Xing et al. developed a trace metal detection test suitable to reveal metal traces transferred from hands to porous surfaces [3, 4]. Other authors demonstrated the enrichment of fingermarks in metal nanoparticles after touching metal and other inorganic objects (namely, firearms, ammunitions, and party sparklers) [5] using techniques based on synchrotron light.

The presence of metal traces on skin or other surfaces can be assessed using SEM–EDS (scanning electron microscope equipped with energy‐dispersive spectroscopy) analysis [6]. The detection of transferred metal particles could be extremely useful in a forensic setting.

The direct transfer of metal particles from the weapon (knife) to skin stab wounds is well known and was demonstrated in real cases using SEM–EDS [7]. The transfer of particles by fingertips also regards gunshot residues. Previous studies demonstrated that gunshot residues can be transferred through handshakes from the hands of the shooter to another individual who was not close to the place where the gun was discharged [8]. The same study also showed that gunshot residues could be transferred through contact with the recently discharged firearm.

Collectively, the literature in this field reveals, on one hand, the potential usefulness of the transferred trace detection, but also its complexity. The analysis of metal traces attributable to transfer on a crime scene represents a hard challenge, due to the many factors that can influence the detection and the interpretation of such traces: among many others, the characteristics of the involved materials (both of the touched item and the surface on which the transfer takes place) and the many events that can alter the crime scene [2]. However, the results obtained so far encourage us to perform new studies in order to understand more about the quality and quantity of primary and secondary transfer of metal particles after touching metal items.

To our knowledge, a combined qualitative and quantitative assessment of metal traces transfer in an experimental setting using SEM–EDS has never been performed.

This pilot study aims to evaluate the persistence of metal residues on fingertips following contact with various objects, as well as the extent to which these residues are transferred from metal surfaces to different materials (skin, glass, and plastic) via human touch. The study was designed to address the following research questions:

1. If one touches a metal item, does it leave any trace on the individual's fingertips (primary transfer)? If yes, in what quantities?
2. Can such metal traces be transferred to other surfaces/other individuals' skin (secondary transfer)? In what quantities?
3. Do the characteristics of the metal item's surface or the recipient surface influence the abundance of particles detected after primary and secondary transfer?

## 2 MATERIALS AND METHODS

**FIGURE 1:**
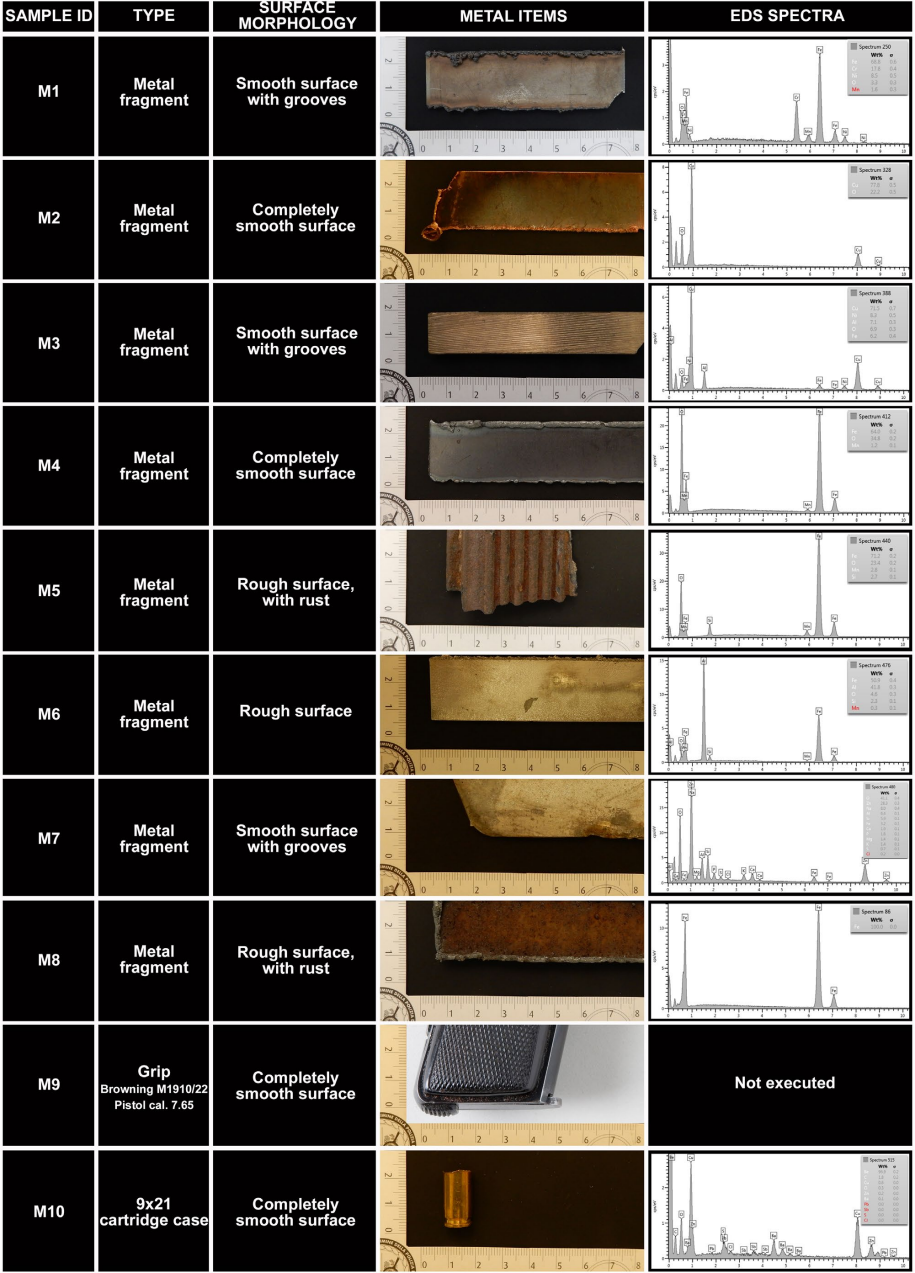
The metal items used in this study. A description of the surface morphology and an image of each item is also reported, as well as the reference spectrum for each item (excluding the pistol, which was not analyzed in order to preserve its integrity).

The study was conducted using 10 metal items (M1–M10) (Figure 1). A fragment of item 1, 2, 3, 4, 5, 6, 7, 8 was severed using a suitable saw from the object. The whole 9 × 21 cartridge case (M10) was examined under SEM–EDS. Instead, regarding M9 (a Browning M1910/22 Pistol cal. 7.65), we preferred not to detach a fragment in order to preserve the integrity of the pistol. A summary of the characteristics of each item is reported in Figure 1, where also a macroscopic photograph of each item is included. Before the experiments, the surface of the metal items was cleaned using disposable tissues in order to remove rust and possible deposited dirt. Experiments were conducted by two people (person 1 and person 2). Thirty minutes before each trial, both of them washed their hands with Marseille soap and water. One control stub was collected from both thumbs of person 1 and from each surface used for the second touch experiment (respectively, glass, plastic, skin of person 2).

### 2.1 First touch samples

Person 1 touched the 10 metal items, applying pressure for 15 s, without rubbing, using both thumb fingertips. For each item, a single fingermark was immediately collected using a metal stub covered by a carbon tape and submitted to the SEM–EDS analyses. Therefore, 10 first touch samples were collected.

### 2.2 Second touch samples

Person 1 touched the 10 metal items (in different areas compared with the first experiment), applying pressure for 15 s, without rubbing, using both thumb fingertips. Then, person 1, after touching each metal item, touched one of three different surfaces: glass, plastic, or skin of a second volunteer (person 2). For each item, one sample was immediately taken from each surface (using a metal stub covered by a carbon tape), obtaining, in total, 30 s touch samples.

### 2.3 SEM–EDS analyses

The analyses were carried out using the scanning electron microscope EM30X (COXEM), equipped with an EDS microprobe (Oxford‐INCA).

**FIGURE 2:**
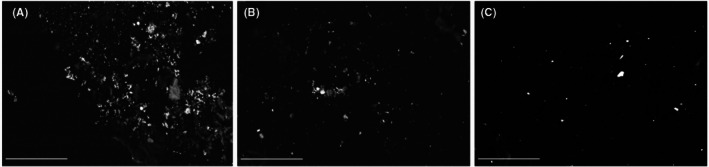
Representative images of the three types of appearance of particles on samples (A) large aggregated residues in the first touch sample M5, backscattered electrons (BSE); (B) small aggregated residues in the first touch sample M4, (BSE); (C) scattered residues in the first touch sample M2 (BSE); scale bar: 50.0 μm.

For each touch sample, a number of selected microscopic fields along six parallel strips were observed at low magnification (1000×) to cover a total area of 2.4 mm^2^ (total number of fields analyzed = 90). The observations were carried out using mainly backscattered electrons (Figure 2).

**FIGURE 3:**
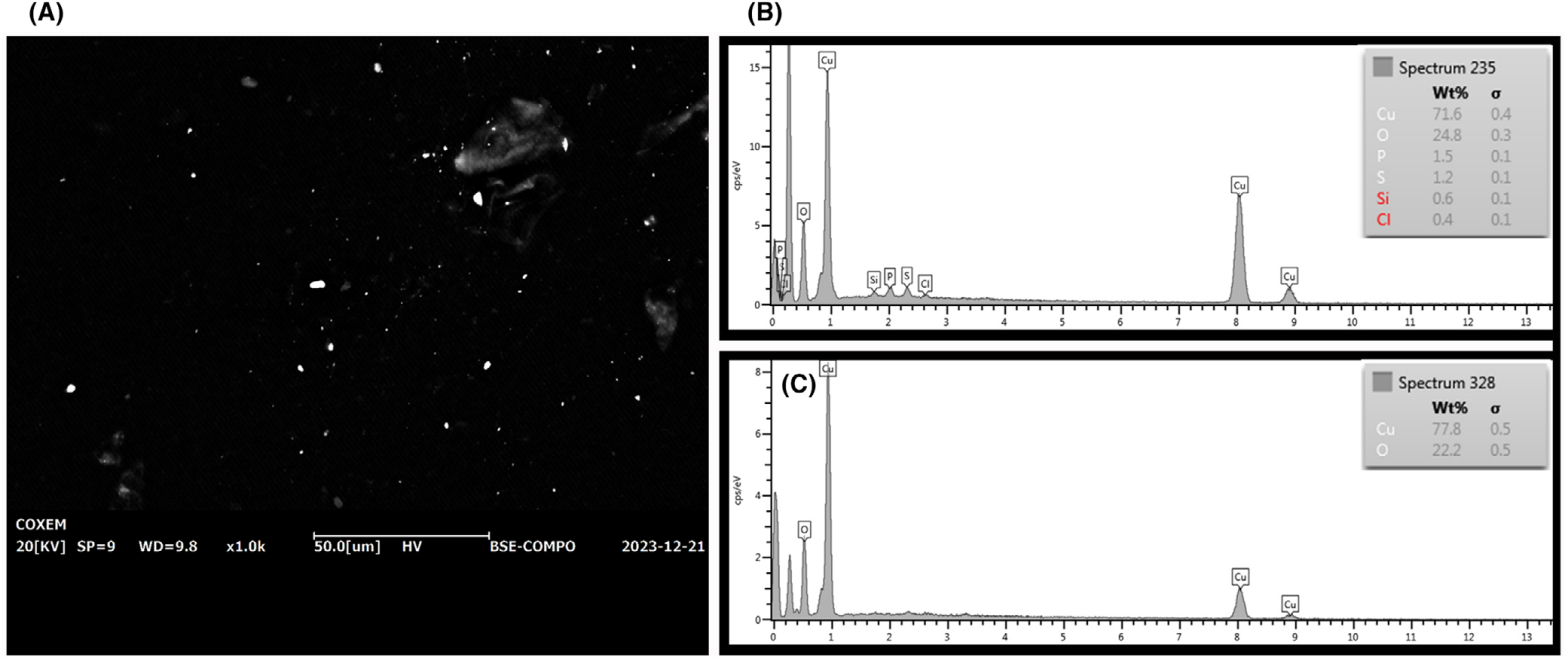
Reference EDS spectra of METAL 2 (A); the corresponding particle spectrum on primary transfer sample (B); the corresponding spectrum from secondary transfer on plastic (C).

Fragments of the metal items were firstly analyzed by SEM–EDS in order to identify the elements characteristic of each item. Iron (Fe), copper (Cu), aluminum (Al), chromium (Cr), manganese (Mn), zinc (Zn), and titanium (Ti) were observed in the metal items M1‐M8. For M9, iron (Fe), lead (Pb), copper (Cu), and zinc (Zn) were considered to give a positive result, based on the well known composition of the pistol's grip [5]. For M10 (the 9 × 21 cartridge case), particles were identified as originating from the item when their EDS spectra contained the characteristic elements previously observed in the reference sample—namely, copper (Cu), zinc (Zn), lead (Pb), barium (Ba), and antimony (Sb). To identify particles as belonging to a specific metal object, the EDS spectra obtained from each particle must contain all the characteristic elements found in the corresponding reference spectra. For example, to attribute a particle to Metal 2 (brass), both Zn and Cu must be present in its EDS spectrum (Figure 3).

The residues detected in each field were analyzed by EDS at a magnification suited to the size of the particle (generally between 5000×–15,000×) and the EDS spectra were compared with those acquired from the fragments of the metal items (reference spectra). Because manual particle counting is extremely time‐consuming and impractical given the heterogeneous and often aggregated distribution of transferred metal particles, we standardized our quantification by examining the same number of fields of view (90) at fixed magnification for each sample, following a consistent observation protocol defined by the SEM software axes. A sample is considered positive if more than five out of the 90 observed fields contain at least 1 particle each that match the reference spectra for each metal object.

Each touch sample was then described by the number of positive fields and the usual distribution of the residues on the sample (i.e., scattered residues, small or large aggregated residues).

## 3 RESULTS

**FIGURE 4:**
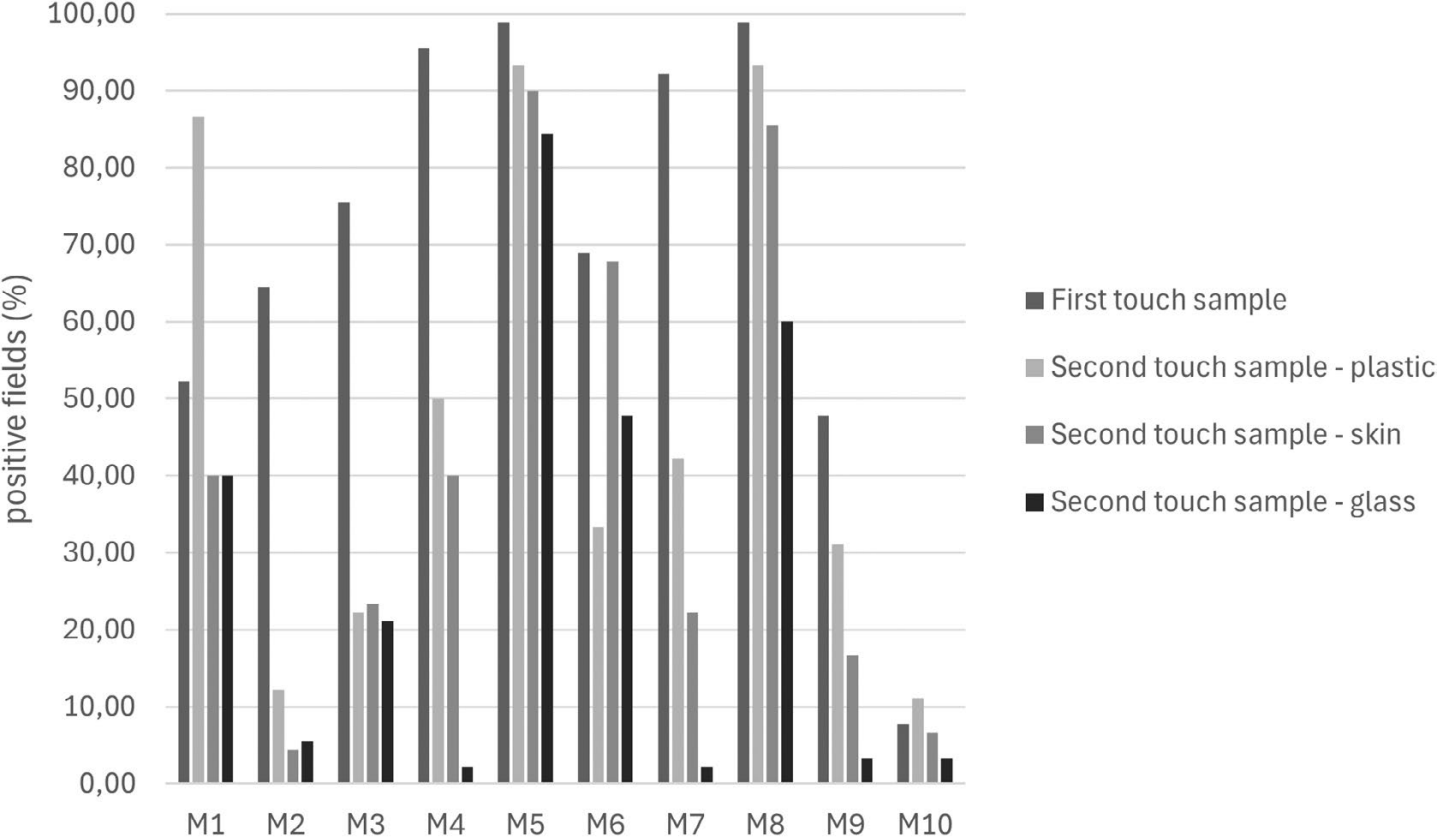
Representation of the number of positive fields reported as percentage observed for the first (dark gray) and second touch samples (plastic surface in light gray, skin surface in medium gray, and glass surface in black).

In the 2.4 mm^2^ area of the touch samples, metal residues containing elements previously observed in the reference items (Figure 1) were detected in all the first and second transfer samples. In the first touch samples, 50% or more positive fields were observed in most of the samples (mean ± SD = 70.22 ± 28.98%, min‐max = 7.78–98.89%). In five samples, more than 75% of the observed fields were positive (samples M3, M4, M5, M7, and M8) and in four of them more than 90% fields were positive (samples M4, M5, M7, and M8). The lowest percentages were instead observed for the firearm and cartridge samples (samples M9 and M10; Table 1, Figure 4). None of the control samples tested positive. Specifically, the first touch control sample showed no detectable metal particles. In the second touch control samples (Figure S1), a few particles were observed and analyzed—namely, one fragment on skin, one on glass, and three on plastic. However, the number of particles detected in these controls was insufficient to consider any of the samples positive.

**TABLE 1:** Positive fields reported as percentage observed in the first and second touch samples.

	M1	M2	M3	M4	M5	M6	M7	M8	M9	M10
First touch sample	52.22% (3)	64.44% (1)	75.56% (2)	95.56% (2)	98.89% (3)	68.89% (1)	92.22% (1)	98.89% (2)	47.78% (1)	7.78% (1)
Plastic	86.67% (1)	12.22% (1)	22.22% (1)	50.00% (1)	93.33% (1)	33.33% (1)	42.22% (1)	93.33% (1)	31.11% (1)	11.11% (1)
Skin	40.00% (1)	**4.44% (1)**	23.33% (1)	40.00% (1)	90.00% (1)	67.78% (1)	22.22% (1)	85.56% (1)	16.67% (1)	6.67% (1)
Glass	40.00% (1)	**5.56% (1)**	21.11% (1)	**2.22% (1)**	84.44% (1)	47.78% (1)	**2.22% (1)**	60.00% (1)	**3.33% (1)**	**3.33% (1)**

*Note*: Within brackets, the global residue distribution is reported (1: scattered residues, 2: small aggregated residues, 3: large aggregated residues). Samples not considered positive (i.e., with fewer than five positive fields) are shown in bold.

When considering the average number of positive fields, calculated using only samples with more than 5 positive fields, no substantial differences were observed among the three surfaces. (Table 1, Figure 4). Namely, the plastic surface showed, on average, 47.56% of positive fields (SD = 32.40%, min–max = 11.11–93.33%), while skin and glass showed, respectively, 43.58% and 50.67% of positive fields (SD (skin) = 30.58%, min–max (skin) = 6.67–90.00%; SD (glass) = 23.58%, min–max (glass) = 21.11–84.44%). Considering the samples defined as negative according to the established threshold (i.e., samples with fewer than 5 positive fields), plastic showed, on average, more positive fields compared with skin (47.56% vs. 39.66%) and skin showed more positive fields compared with glass (39.66 vs. 26.99%).

Considering the surface morphology of the metal items (smooth or rough surface), no evident differences were observed in the number of particles on the first touch samples. Similarly, no clear differences between second touch samples on plastic were revealed with respect to the surface morphology of the metals. On the other hand, glass and skin second touch samples showed, respectively, always more than 45% for rough items and less than 40% positive fields for smooth items. In particular, most of the glass second touch samples from smooth items were below the positivity threshold (Figure 4).

On the whole, as shown in Figure 4, even though quite variable, the amount of transferred particles appears to be higher for metal items with a rough surface compared with smooth ones. The difference in transfer according to the surface morphology is more marked considering the second touch samples.

Then, we classified each sample according to the appearance of the observed particles into large aggregates, small aggregates, and scattered particles (Figure 2). Aggregations of particles were observed mainly in five first touch samples, as large aggregations in samples M1 and M5 and small aggregations in samples M3, M4, and M8. In the remaining samples, mostly scattered residues were detected (Figures 1 and 4). No relationship was detected between the appearance (according to spatial distribution) of particles and surface morphology of the metal items.

## 4 DISCUSSION

The present results confirm that, after touching a series of different metallic items, traces of them persist on the fingertip's skin and can be detected using SEM–EDS. Moreover, such traces can be transferred by touching a second surface and detected using the same technique. Despite the variability between the different samplings (also reported in previous studies [9]), the assessment of the transferred particles revealed, as expected, a tendency to a decrease in the amounts of particles between the first and the second touch, regardless of the touched surface (plastic, skin or glass).

While our findings indicate a general decrease in the amount of transferred metal particles between the first and second touch, this trend was not observed for all items. Notably, for M1 and M10, the percentage of positive fields recorded for the second touch on plastic was higher than for the first touch (86% vs. 52%), and for M5 and M8, the differences between first and second touch on plastic were minimal and may not be statistically significant. These exceptions, which are apparent in Table 1, suggest that individual or item‐specific variability may play a role, and highlight the importance of further studies with larger sample sizes and repeated measures to confirm these findings and understand their reproducibility. Differences in the number of particles released after the second touch were assessed, revealing the highest amount of particles on plastic, followed by skin and glass, for most metal objects. The amount of transferred particles is higher for metal items with a rough surface compared with smooth ones.

This work provides experimental data that can be useful in a forensic setting, in order to link objects, actions, and people. For example, at a crime scene, the detection of metal particles within fingermarks attributed to a specific individual can provide direct evidence of whether that person has handled an object of forensic relevance, thereby assisting in the reconstruction of crime dynamics. This approach can also help to establish if a victim has been touched by someone who previously held a weapon or another metal object found on the scene. Moreover, the metal trace analysis can be useful in order to make a comparison between a known object (as the crime weapon) and residues on the body or on the clothes, where the sampling can be guided by the previous fingermarks detection. Fingerprints detection can be used as a guide to sampling in order to get to know which surface has been touched by the same person who previously handled a metallic weapon. As the amount of metal particles decreases touch by touch, the concentration of particles on each fingerprint can tell us something about the order of touching [2]. This can be useful in understanding the crime dynamics and reconstructing the criminal action.

However, it must be taken into account that the present study was carried out in an experimental setting that mimics as much as possible real‐world situations, but in caseworks many more variables play a role and it is difficult to take into account all of them. Indeed, not only the source material, but also the recipient surface, the strength and the duration of the contact have been reported to potentially influence the extent of traces transfer [5]. Moreover, the time between the touching of an item and the collection can influence the quantity of particles which remain on one's fingertips. The individual variability of the metal transfer, depending on possible subject's characteristics that make one a “good” or “bad” transferrer, must be investigated with further experimental studies. In order to improve the applicability of such methods, indeed, the probability of being a “good” particle carrier should be clarified, as well as the individual characteristics that can influence the presence of metal particles on fingertips.

In addition to the confounding factors previously described, the detection of transferred metals may also introduce interpretive challenges. For instance, because metallic particles can be redistributed through skin‐to‐skin contact, a person who has handled a weapon may inadvertently transfer these particles to others via touch, resulting in the presence of metal traces on individuals who were not directly involved in the criminal act. It is important to emphasize that the detection of metal particles on an individual's skin or fingermarks does not, on its own, constitute definitive evidence of direct contact with a specific object. Many metals, such as those found in stainless steel and copper alloys, are widespread in domestic and occupational environments, and their particles can be introduced to the skin through a variety of mundane activities, including cooking, handling tools, or working with metal surfaces. In this study, all volunteers thoroughly washed their hands before each trial, and no detectable metal particles were observed in the control samples; however, in real‐world circumstances, the background level of metal particles on the skin could be substantially higher, especially among individuals engaged in certain professions or activities (e.g., electricians, plumbers, culinary work). Therefore, the evidentiary value of detected particles must be carefully interpreted in the context of potential environmental and lifestyle exposures. Future studies should aim to characterize typical background levels of environmental metal particles across diverse populations, as these background contaminants may limit the specificity of trace evidence based on commonly encountered metals.

Another issue is related to the technique which is used to identify the metal traces.

In some of the previous studies about this topic, the authors tried to identify metal traces using colored or fluorescent indicators (e.g., trace metal detection tests, TMDTs) [4]. Such techniques have strong limitations, essentially related to the high rate of false positives and the low specificity, as a consequence of the unsuitability of TMDT to identify the real composition of the detected trace. Other authors used more sophisticated techniques, based on synchrotron radiation, such as X‐ray fluorescence microscopy and X‐ray absorption near‐edge structure (XANES), in order to investigate the transfer and the persistence of trace elements in fingerprints. The results showed that the fingerprints presented Fe and Pb after handling a gun barrel and Cu and Zn after handling an ammunition cartridge. However, the methods used by Hackett et al., based on synchrotron radiation, are not easily available for forensic practitioners, requiring access to a synchrotron facility. On the other hand, SEM–EDS approach, used in the present work, is available in many Forensic Pathology Institutes and much less time‐consuming. Moreover, the sampling is easy and quick, as only a common stub with carbon tape is required. With SEM–EDS, it is possible to analyze the elemental composition of a specific object or weapon and directly compare its reference spectra to the particles detected on a suspect's hands or on surfaces from the crime scene. Additionally, the use of backscattered electron imaging facilitates the rapid identification of metal fragments, which appear markedly brighter against the darker background, enhancing the contrast and specificity of trace detection. Then, those particles can be analyzed with EDS in order to know the elemental composition of detected particles. What SEM–EDS cannot provide is the distinction between metal Iron (Fe) and ferric iron Fe3+, that serve to distinguish metal particles from, for example, hemoglobin. However, this distinction is possible only using very advanced techniques based on synchrotron light.

In conclusion, the present experimental work supports the hypothesis that metal particles can be transferred not only to the fingertips of the person who touches something, but also to secondary surfaces, and such traces can be detected and identified successfully using SEM–EDS. The nature of the second surface, as well as to a lesser extent the characteristics of the metal object surface, can influence the quantity of traces which can be detected. These results encourage further research on this field, aiming to understand more about the possible reproducibility of the patterns of transfer and their applicability to crime reconstruction of real cases. Future studies are needed to systematically assess the persistence of transferred metal traces on skin over time, as rapid loss or removal of these particles could significantly limit their reliability and value as forensic evidence.

## CONFLICT OF INTEREST STATEMENT

The authors have no conflict of interest to disclose.

## Supporting information


Figure S1.

## Data Availability

The data that support the findings of this study are available from the corresponding author upon reasonable request.
