# Supplementary figures and images for: Qualitative and semi‐quantitative primary and secondary transfer of metal traces by human touch detected using SEM–EDS technique: A pilot study
Source: J Forensic Sci. 2026 Feb 11;71(3):1420–7. doi: 10.1111/1556-4029.70285 (PMC13139813; doi:10.1111/1556-4029.70285)

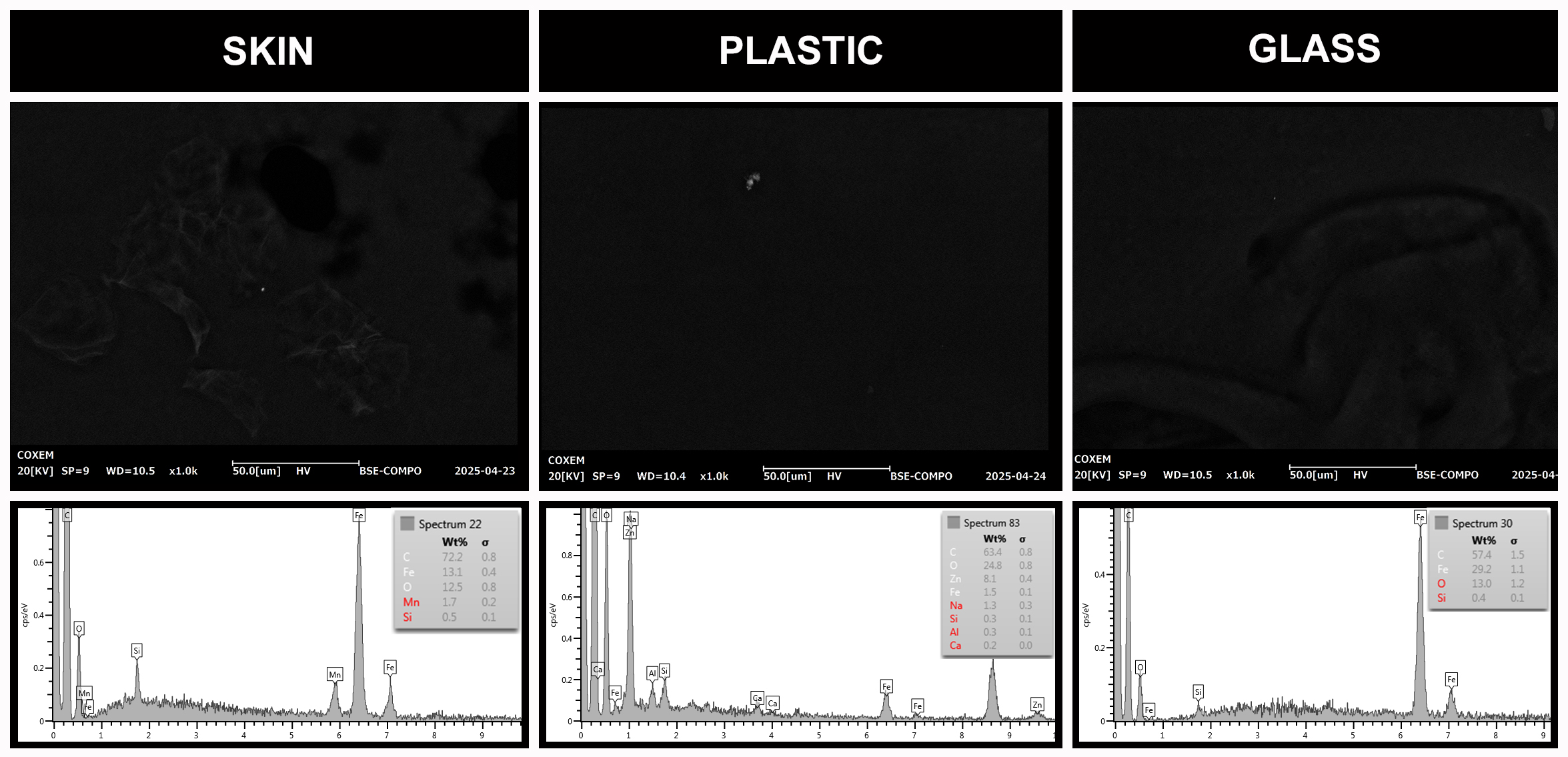

Supplement: Supplementary file 1 — Figure S1. [file JFO-71-1420-s001.jpg]
